# Where to Settle—Settlement Preferences of *Mytilus galloprovincialis* and Choice of Habitat at a Micro Spatial Scale

**DOI:** 10.1371/journal.pone.0052358

**Published:** 2012-12-14

**Authors:** Christina Carl, Andrew J. Poole, Mike R. Williams, Rocky de Nys

**Affiliations:** 1 School of Marine and Tropical Biology, Centre of Sustainable Tropical Fisheries and Aquaculture, James Cook University, Townsville, Australia; 2 CSIRO Materials Science Engineering, Belmont, Australia; 3 Fisheries Victoria, Fisheries Research Branch, Queenscliff, Australia; 4 Victorian Shellfish Hatcheries Pty Ltd, Queenscliff, Australia; University of New South Wales, Australia

## Abstract

The global mussel aquaculture industry uses specialised spat catching and nursery culture ropes made of multi-filament synthetic and natural fibres to optimise settlement and retention of mussels for on-growing. However, the settlement ecology and preferences of mussels are poorly understood and only sparse information exists in a commercial context. This study quantified the settlement preferences of pediveligers and plantigrades of *Mytilus galloprovincialis* on increasingly complex surfaces and settlement locations at a micro spatial scale on and within ropes under commercial hatchery operating conditions using optical microscopy and X-ray micro-computed tomography (µCT). *M. galloprovincialis* has clear settlement preferences for more complex materials and high selectivity for settlement sites from the pediveliger through to the plantigrade stage. Pediveligers of *M. galloprovincialis* initially settle inside specialised culture ropes. Larger pediveligers were located close to the exterior of ropes as they increased in size over time. In contrast, smaller individuals were located deeper inside of the ropes over time. This study demonstrates that X-ray µCT is an excellent non-destructive technique for mapping settlement and attachment sites of individuals as early as one day post settlement, and quantifies the number and location of settled individuals on and within ropes as a tool to understand and optimise settlement in complex multi-dimensional materials and environments.

## Introduction

The genus *Mytilus* with two key species, *Mytilus galloprovincialis* and *M. edulis*, is the major contributor to the mussel aquaculture industry [1], [2], and is worth more than 1.2 billion USD per annum [3]. Mussel seed for this industry is either collected from the wild using spat catching ropes, or alternatively collected from drift macroalgae and natural mussel beds. In addition, mussel seed is produced in closed life-cycle hatchery culture [4]. The success of the global mussel aquaculture industry relies heavily on high settlement rates and retention of mussel spat on ropes for on-growing [5]. To optimise settlement and retention, specialised spat catching and culture ropes made of multi-filament synthetic and natural fibres are used [6]–[10]. However, little is known about the fundamental drivers for larval selection of preferred settlement sites for attachment on materials for commercial species of mussels. Understanding these drivers is vital to develop and implement mechanisms to optimise and manage the settlement of mussels, and ensure a sustainable practice in the mussel aquaculture industry.

In contrast to many sessile invertebrate larvae, mussel settlement is not permanent and individuals of *Mytilus* can detach and re-settle in an alternative habitat [1], [11]. Larvae of *M. galloprovincialis* initially settle as pediveligers and actively explore the substratum by crawling with a foot [1], [12], [13], which is a complex muscular and glandular organ with cilia, also used for byssus secretion [1], [2]. Pediveligers are capable of discriminating between different substrata [1] and are selective in their preference of settlement sites [6], [8], [9], [14]. If the substratum is unsuitable, they can withdraw their foot and swim off [15]. In these circumstances metamorphosis can be delayed for several weeks [16] and pediveligers repeat the exploratory pattern of swimming and crawling behaviour until a suitable settlement site is found [1]. If a suitable substratum is found, an adhesive plaque and byssal thread is deposited onto the surface [17]. Following metamorphosis, post-larvae are termed plantigrades [15], spat, seed or juveniles [18]. Anecdotal observations at mussel hatcheries suggest that pediveligers initially settle inside the complex structure of multi-filament ropes, supporting an influence of topography on initial settlement preference in large-scale commercial production.

In laboratory based studies, topographic features have a demonstrated role in settlement choice of pediveligers and this effect is far greater than chemical cues [19] and wettability [20]. Textured surfaces provide an attractive surface for larval settlement with a high surface area to volume ratio, and crevices provide high quality refuge from hydrodynamic forces, drifting objects, and predation [21]. This reduces post-settlement mortality [8], [21]. In general, mussel settlement is lower on smooth surfaces, with topographic features strongly enhancing settlement [19], [20], [22]. Attachment is also more rapid on surfaces with topographic features [1], including branching algae [23], shells [14], and hydroids [23], with fine-branching substrata being preferred over medium- and coarse branching materials [23]. Furthermore, mussels actively select favourable sites and higher numbers of spat attach to node areas of substrata than to inter-node areas regardless of the branching of substrata [23], while the absence of filamentous substrata prolongs the larval stage and delays metamorphosis [24]. More specifically, preferences can be defined to accurate sizes, with a topographic width of 400 µm being a preferred initial settlement site for pediveligers of *M. galloprovincialis* [20]. Importantly, any changes in preference as pediveligers grow remain undefined.

In contrast to pediveligers, adult mussels settle on all types of firm substrata [25] to form dense mussel beds [1], suggesting that the effect of topography as a settlement cue becomes less important for adult mussels. However, there has been no effort to deconstruct the direct effect of microtopography on the settlement of mussels beyond the metamorphosis of pediveligers.

Using the combination of manipulative studies and quantitative measurements, the aim of this study is to determine settlement preferences and locations of *M. galloprovincialis* under commercial hatchery operating conditions. Firstly, settlement preferences of pediveligers and plantigrades are quantified for surfaces with increasing complexity. Secondly, in an applied approach, the micro spatial scale settlement of pediveligers on and within culture ropes from a commercial mussel hatchery is quantified and mapped for 16 days post initial settlement using optical microscopy and X-ray micro-computed tomography (µCT).

## Materials and Methods

### Culture of pediveligers and on-growing

Approximately 100 adult mussels of *M. galloprovincialis* were collected as broodstock (F2) from Clifton Springs Aquaculture Fisheries Reserve, Victoria, Australia, in April 2012 and transported to the Victorian Shellfish Hatchery in Queenscliff, Victoria, Australia. Detailed spawning procedure and larvae production procedures are provided in Pettersen et al. [26]. The hatchery operated under the permit RP 943 obtained by Fisheries Victoria. No specific permits were required for the described laboratory studies.

Larvae were reared in 9 aerated flow-through tanks, each holding 400 L filtered seawater (FSW; 1 µm and UV sterilised) with an initial flow rate of 1.7 L min^−1^. The flow rate was increased to 2.5 L min^−1^ after 5 days post fertilisation, and to 3.3 L min^−1^ after 8 days post fertilisation. The larvae were maintained at a density of 25–40 larvae mL^−1^ at 16°C under constant light. Larvae were fed *Chaetoceros calcitrans* (CS-178), *Pavlova lutheri* (CS-182), and *Isochrysis galbana* (clone T.ISO, CS-177) daily in equal parts at a target background residual cell density of 50,000 cells mL^−1^. After 12 days post fertilisation, *C. muelleri* (CS-176) was added to the diet (33% *I. galbana*, 33% *P. lutheri*, 11% *C. calcitrans*, 22% *C. muelleri*). The water in all tanks was changed every other day.

The pediveliger stage was reached at 22 days post fertilisation and larvae were competent to settle. In the conditions used for settlement and on-growing under hatchery production, reproduced in assays below, pediveligers of several larval rearing tanks were combined until the required number of approximately 19.3 Mio larvae was achieved for transferring these into one of six nursery tanks at a density of approximately 4.8 pediveligers mL^−1^ in a closed culture system. Each nursery tank held approximately 4000 L FSW and 750 m of spat catching ropes (150 ropes, each 5 m) as a settlement surface. The outdoor nursery tanks were aerated and covered with a shade cloth to reduce the growth of filamentous algae and to minimise water temperature fluctuations. The water was completely renewed every second day. Mussels were fed daily at a target cell density of 50,000 cells mL^−1^ of *C. muelleri* (50%), *P. lutheri* (25%), and *I. galbana* (25%).

### Laboratory settlement assays

To quantify the effects of surface complexity on the settlement of *M. galloprovincialis*, three settlement surfaces with increasing complexity were tested. These were smooth polypropylene rods (Gehr GmbH, Germany), textured polypropylene rods with a square wave profile of 400 µm (Gehr GmbH, Germany), and multi-filament braided polypropylene nursery ropes (Whittam ropes, Australia) used by local mussel farmers in Port Phillip Bay, Victoria, Australia. Polypropylene rods were textured by cutting a 400 µm wide and 400 µm deep thread into the rod, with a spacing of 400 µm between the thread. This feature size (400 µm) is highly effective in enhancing the settlement rate of pediveligers of *M. galloprovincialis* [20]. Each test surface (rods and rope) was cut to a length of 100 mm. The rods and rope had a diameter of approximately 20 and 12 mm, respectively. To suspend the test surfaces in water, a hole was drilled through the top end of each polypropylene rod and fishing line inserted. Similarly, fishing line was inserted into the top end of each braided rope for suspension. To ensure full suspension of each test surface in the settlement assays, a stainless steel weight (∼19 g) was glued (Silkaflex-PRO, Silka) to the bottom end of each test surface.

Mussels were collected for settlement assays at 22 (pediveliger stage), 30, and 38 days post fertilisation (plantigrade stage) from the Victorian Shellfish Hatchery. Pediveligers were collected from a larval rearing tank approximately 1 h prior to the transfer to nursery tanks. Plantigrades were collected off a rope from a designated outdoor nursery tank. First, to conduct settlement assays with no choice for *M. galloprovincialis* between polypropylene settlement surfaces, one each of the three surfaces (*n* = 10) were suspended individually in glass beakers filled with 900 mL FSW. Each test surface was suspended from a small rod using fishing line. The rod was placed horizontally on the top of the beaker, with the test surface immersed vertically in the water. The water was aerated using glass pipettes to ensure an even suspension of mussels in the water column. Approximately 100 individual mussels (pediveligers or plantigrades) were placed in each glass beaker and maintained in a temperature controlled room at 17°C in a 12 h light:12 h dark cycle. The settlement of individuals on all surfaces including the beaker was measured after 48 h. Test surfaces were dipped three times in the water column to remove unattached mussels. Subsequently, these and other mussels suspended in the water column were captured on a 100 µm sieve and counted. Mussels attached to smooth and textured polypropylene rods, and glass beakers, were counted using a dissecting microscope (Olympus SZX7). To count the number of mussels attached to the ropes, these samples were teased apart and individually rinsed with FSW. The detached mussels were retained with a sieve (100 µm) and subsequently counted. Second, to conduct multiple choice settlement assays using *M. galloprovincialis*, the three surfaces were randomly attached to a rod and suspended in a glass beaker (*n* = 10) as previously described. Approximately 100 individual pediveligers or plantigrades were added to each beaker and maintained in the same room. After 48 h, the number of settled and unattached mussels was recorded.

### Micro spatial scale settlement onto ropes under hatchery conditions

#### Rope samples in small-scale nursery tanks

To determine the settlement at a micro spatial scale onto and within culture ropes under commercial hatchery operating conditions, a total of 468 test rope pieces (Whittam ropes, Australia) were submersed in three independent outdoor tanks at the Victorian Shellfish Hatchery in Queenscliff. Each tank held 156 rope pieces and was a small-scale of the commercially used nursery tanks at the hatchery described previously. Each test rope piece had a length of 150 mm and was tied to the bottom and the top of the tank using fishing line so that all rope samples were immersed vertically in the water at a depth of approximetaly 130 mm below the surface. Each small tank was filled with 130 L FSW and approximately 20,000 pediveligers (22 days post fertilisation) as determined by subsampling were added to each tank. The feeding regime, density of larvae and rope per unit volume were equal to hatchery standards for the commercial tanks (see *Culture of pediveligers and on-growing*). The water was completely renewed in each tank after 24 h and therefore all non-settled larvae were also removed from the tanks after 24 h. More than 99% of pediveligers settled within this time and were no longer suspended in the water column. Sets of ropes (*n* = 3) were collected from each tank one day after settlement (23 days post fertilisation) and subsequently on every fourth day for 16 days (39 days post fertilisation). Within each rope, from each set, a section of 100 mm within the centre of the rope was marked using cable ties and the number of settled mussels within the marked section counted using a dissecting microscope (Olympus SZX7). Subsequently, mussels were removed from the outside of the rope using tweezers, preserved in 70% ethanol and images were taken for size measurements (Image J). The assayed ropes were then discarded.

#### Rope samples in individual containers

To quantify the movement of mussels from the inside of the rope outwards as individuals grow larger, a total of 15 rope pieces were collected from each small-scale nursery tank (3 tanks; 45 rope pieces in total) after one day post settlement (23 days post fertilisation). The ropes were then placed individually into independent labelled containers, each holding 1500 mL of FSW. Notably, ropes in individual containers were not subject to longer term larval supply and provide a contrast to rope samples maintained in small-scale nursery tanks as movement between ropes was excluded. This confirms that all subsequent mussels are a product of the initial settlement of pediveligers at 23 days post fertilisation. The water in each container was gently aerated and the containers were maintained at 17°C with 12 h light:12 h dark cycle. The feeding density was maintained at 50,000 cells mL^−1^ as described previously for nursery tanks. The water and holding containers were changed every other day. To determine the initial settlement and subsequent movement of mussels on the ropes over time, one set of ropes (*n* = 3) from each small-scale tank was removed and assayed on 1, 5, 9, 13, and 17 days post settlement by quantifying the number of settled mussels on the outside of the rope within each marked 100 mm section as previously described. All mussels were removed using tweezers, counted and preserved in 70% ethanol for size measurements. To minimise stress and avoid repeated sampling, all assayed ropes were discarded.

#### Imaging micro spatial habitat with X-ray µCT

**Figure 1:**
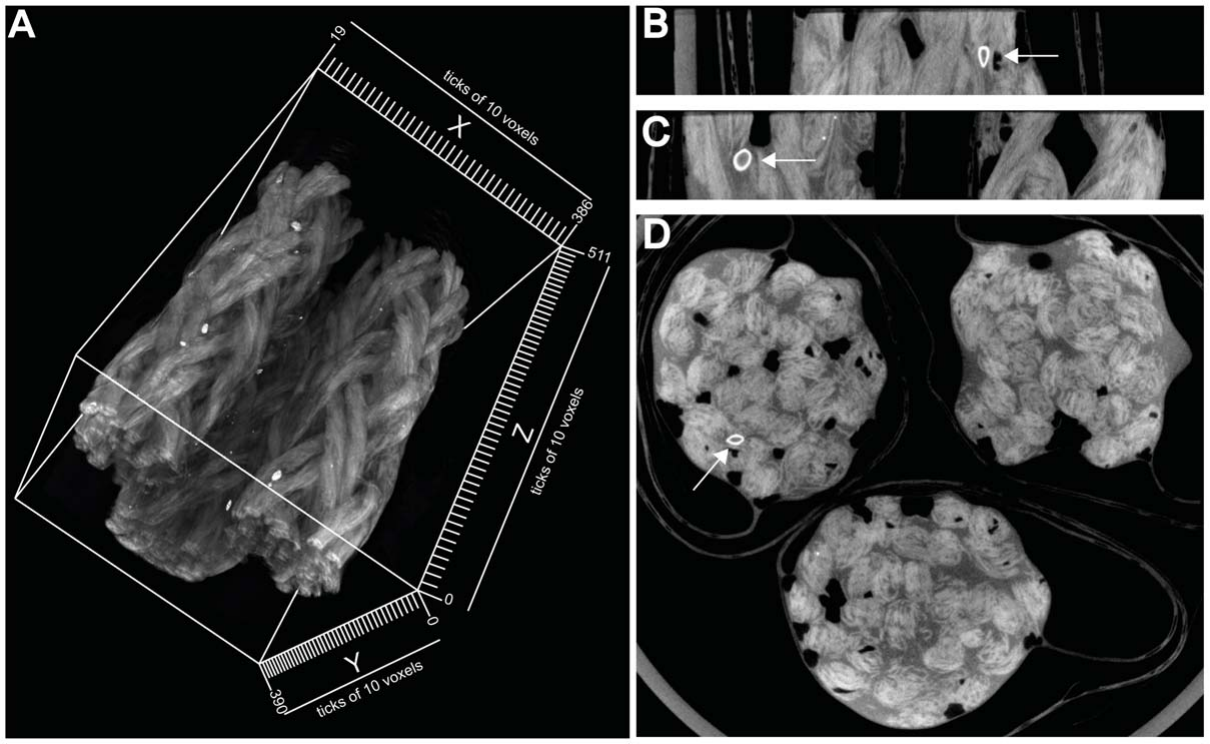
Three-dimensional tomogram of rope samples collected 17 days post settlement. (A) Tomogram with a reduced volume of 512^3^ voxels and a voxel size of 78 µm for visualising purposes. (B) Micro spatial scale settlement of a mussel in x-plane, (C) y-plane, and (D) z-plane. Arrows indicate a mussel, which is clearly distinguishable by the oval shape with a hollow centre, which characterises the two shell valves. Tomograms with 1024^3^ voxels were generated and used for analysis with a voxel size of approximately 39 µm.

In addition, the specific settlement locations and the depth of settlement within the rope were qualitatively determined using X-ray µCT. This accurately images settlement locations at a micro spatial scale and is a non-destructive method, avoiding the need to prepare and section samples which may cause damage or distortion. One rope piece was collected from each small-scale nursery tank (*n* = 3) on 1, 5, 9, 13, and 17 days post settlement (23, 27, 31, 35, and 39 days post fertilisation). Immediately on collection, all ropes were placed in 70% ethanol for 30 min and subsequently vacuum packed in plastic wrap (Sunbeam VAC 660) to minimise dislocation of mussels during transportation. For X-ray µCT imaging, the samples were transported from the hatchery in Queenscliff to the µCT facility located at the Australian National University, Canberra. The in-house designed X–ray µCT device is built on a 3 m parallel optical rail and consists of a X-ray source (X-Tek RTF-UF225), rotation stage (Newport RV120PP) and X-ray camera (Roper PI-SCX100:2048) (see [27] for details). The location of the rotation stage and the camera are adjustable on the rail, which results in changes of the magnification of the sample. The magnification is determined by the proximity of the sample to the X-ray source, compared with the distance between the camera and the source [28]. For this study, the sample distance was 800 mm, which resulted in a voxel size of approximately 39 µm. A “voxel” is a cubic volume element, which represents a three-dimensional data point in the tomogram. The three-dimensional tomogram (Figure 1A) visualises the structure and variation of composition within the sample and was generated by collecting a series of two-dimensional radiographs, collectively called projection data [27]. In order to collect the projection data at different viewing angles, the sample was rotated through 360°. To minimise the scan time and to stabilise the samples, a total of three rope samples (one sample from each small-scale nursery tank collected at the same time point) were vertically placed side by side in a small plastic container fixed on the rotation stage for each scan. The rope samples were kept vacuum packed and individually labelled using aluminium foil stripes allowing identification in the tomogram. The X-ray source was run with 80 kV at 120 µA to optimise the contrast of the mussel shells in the rope samples. A total of 1440 projections were obtained per revolution and the scan time was 5 h for each scan. Volumes of 1024^3^ voxels were generated and each rope sample was scanned approximately 35 mm in length. To generate a tomogram, the projection data were processed with a Feldkamp reconstruction algorithm [27], [29]. All data processing was carried out on a Compaq AlphaServer super-computer located at the Australian Partnership for Advanced Computing, Australia's national supercomputing facility.

The images from three-dimensional tomograms were visualised and analysed using the in-house developed program “NCViewer”. This program has the feature to visualise the tomogram slice by slice in each plane (x, y, z; Figure 1B–D). The size of each mussel was individually measured in each plane. To measure the settlement depth of mussels within the rope, the distance of each individual to the exterior of the rope was quantified in the z-plane (Figure 1D). The closest point of each mussel was used as a reference point for distance measurements to minimise the effect of shell length on the distance to the exterior of the rope. Uniform distance measurements were obtained using the plastic wrap, in which each rope sample was vacuum-sealed as a reference. Mussels were clearly distinguishable in the tomograms by an oval shape with a hollow centre, which characterised the two shell valves (Figure 1B–D). The contrast of the mussels increased with age as the X-ray density of the calcium carbonate of the shell increased with growth. To quantify settlement depth within ropes among mussel with different sizes, mussels were grouped into eight size classes based on their maximum shell length measured in any of the three planes (x, y, z): 250–349, 350–449, 450–549, 550–649, 650–749, 750–849, 850–949, and >950 µm in shell length.

### Statistical analysis

Data were analysed by one- or two-factor permutational multivariate analysis of variance (PERMANOVA). The Bray-Curtis dissimilarity measure was used for all PERMANOVA's and *P*-values were calculated using unrestricted permutation of untransformed raw data and permutation of residuals under a reduced model with 9999 random permutations for one- and two-factor PERMANOVA's, respectively. If there was a significant difference, pair-wise *a posteriori* comparisons were made among the significant groups using the Bray-Curtis similarity measure (α = 0.05). Statistical analyses were performed using PRIMER 6 (v. 6.1.13) and PERMANOVA+ (v. 1.0.3) [30]. Data are reported as mean ±1 standard error (SE).

The effect of surface complexity on the settlement of *M. galloprovincialis* in no choice and choice assays were considered fixed factors. Beaker was included as a random factor for multiple choice assays as test surfaces in one beaker were not independent. There was no significant interaction term (surface complexity × beaker) as surfaces were not replicated in each beaker. To test for differences in settlement of mussels on the outside of ropes under hatchery conditions over time (in small-scale nursery tanks and individual containers), time (days post fertilisation) was treated as a fixed factor and tank was included as a random factor. There was no significant interaction term. To assess differences in the depth of settlement of mussels within ropes using µCT, PERMANOVAs were performed for each sampling point (days post settlement) with size class as a fixed factor and tank as a random factor. There was no significant interaction term.

## Results

### Laboratory settlement assays

**Figure 2:**
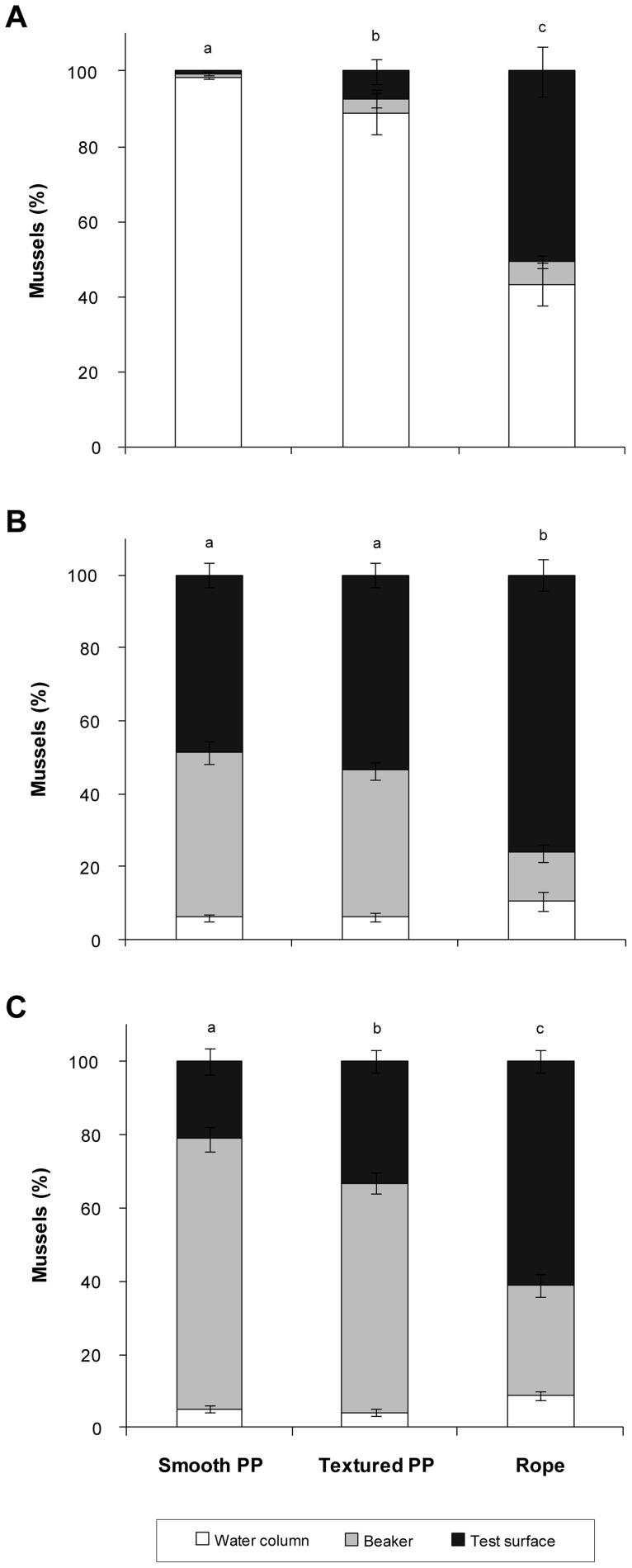
Settlement of *Mytilus galloprovincialis* in settlement assays with no choice between polypropylene (PP) surfaces. Number of mussels (%) settled on test surfaces (smooth PP, textured PP, rope) and glass beaker and suspended in the water column after 48 h. Settlement assays were conducted with mussels (A) 22 days, (B) 30 days, and (C) 38 days post fertilisation. Means ± SE are shown (*n* = 10). Superscript letters indicate significant differences between test surfaces (pair-wise *a posterior* test, α = 0.05).

Increasing complexity of the surfaces significantly enhanced the settlement of *M. galloprovincialis* in all settlement assays with no choice between polypropylene surfaces, regardless of age (Figure 2A–C). Settlement was consistently the highest on rope samples, the most complex surface tested.

For assays using pediveligers (22 days post fertilisation) with no choice between test surfaces, settlement significantly differed among all tested surfaces (one-factor PERMANOVA: *F_(9,27)_*  = 26.23, *P*<0.001; Figure 2A). The lowest number of pediveligers settled on smooth polypropylene (0.8±0.2%) and settlement increased with increasing surface complexity to more than 50% on rope. Consequently, less than 44% of pediveligers were unattached and suspended in the water column after 48 h in no choice assays with rope, while more than 88% of pediveligers were in the water column in assays with smooth and textured polypropylene. Overall, the number of individuals settled on beakers ranged from 0.7±0.3% to 6.0±1.5% pediveligers.

Similarly, surface complexity significantly enhanced the settlement of 30 day old plantigrades (*F_(9,27)_*  = 12.00, *P*<0.001; Figure 2B). Significantly fewer plantigrades settled on smooth (48.6±3.4%) and textured polypropylene (53.6±3.2%) than on rope samples (76.0±4.3). The number of mussels suspended in the water column also decreased with increasing age and ranged from 6.2±1.0% to 10.7±2.6% for 30 day old plantigrades. The lowest settlement of plantigrades on beakers occurred in assays with rope samples (13.4±2.4), while the highest number of plantigrades settled on beakers in assays with smooth polypropylene (45.2±3.3%).

Settlement assays conducted with 38 day old plantigrades were consistent with assays conducted 22 and 30 days post fertilisation, with a significant difference in the settlement of individuals between all surfaces (*F_(9,27)_*  = 16.57, *P*<0.001; Figure 2C). Plantigrades had significantly lower settlement on smooth polypropylene (21.1±3.5%) than on textured polypropylene (33.3±3.2%) or rope (60.9±2.9%). Overall, the number of unattached plantigrades was below 9% in all assays and the settlement of mussels on beakers was higher than on the test surfaces with the exception of rope. Between 62.4±2.8% and 73.9±3.3% plantigrades settled on beakers in assays with textured and smooth polypropylene, respectively. In contrast, 30.1±3.1% of plantigrades settled on beakers in assays with rope.

**Figure 3:**
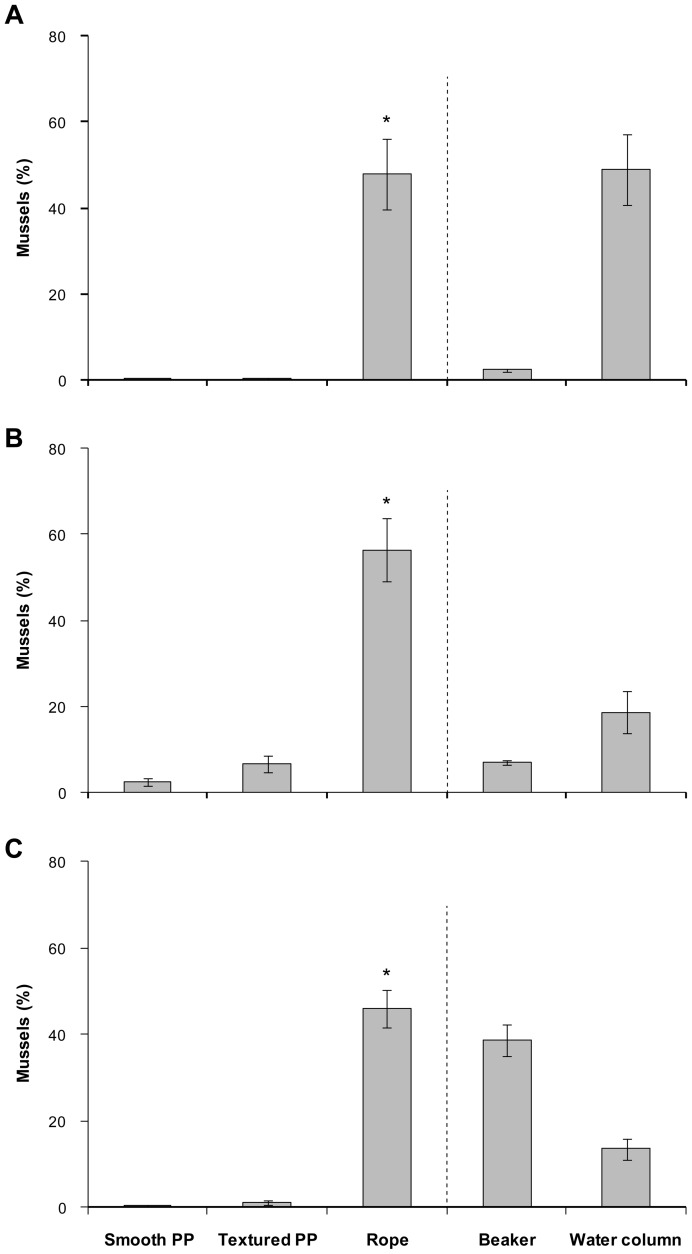
Settlement choice of *Mytilus galloprovincialis* in multiple choice assays. Number of settled mussels (%) on test surfaces (smooth polypropylene (PP), textured PP, rope) and glass beaker and suspended in the water column after 48 h. Settlement assays were conducted with mussels (A) 22 days, (B) 30 days, and (C) 38 days post fertilisation. Means ± SE are shown (*n* = 10). Statistical analyses were only performed for test surfaces (to the left of the dotted line). Asterisk indicates significant differences between test surfaces (pair-wise *a posterior* test, α = 0.05).

For all multiple choice assays, significantly higher numbers of mussels, regardless of age, settled on rope samples, with no significant difference between smooth and textured polypropylene (Figure 3A–C). Broadly speaking, settlement assay where *M. galloprovincialis* had a choice between test surfaces provided similar results to assay with no choice between test surfaces.

For multiple choice assays with pediveligers (22 days post fertilisation), surface complexity significantly enhanced the settlement of *M. galloprovincialis* (one-factor PERMANOVA: *F_(2,18)_*  = 61.40, *P*<0.001; Figure 3A). Less than 1% of pediveligers settled on smooth or textured polypropylene, while 48.1±8.2% pediveligers settled on the rope. Approximately 50% of pediveligers were suspended in the water column after 48 h, whereas only 2.4±0.4% pediveligers settled on the beaker.

In a similar manner to settlement preferences of pediveligers, surface complexity significantly influenced the settlement choice of 30 day old plantigrades (*F_(2,18)_*  = 17.90, *P*<0.001; Figure 3B). The settlement of plantigrades was low on smooth (2.4±0.9%) and textured polypropylene (6.8±2.0%), while the majority of plantigrades settled on the rope (56.4±7.4%). More mussels were suspended in the water column (18.7±4.8%) than attached to the glass beaker (7.0±0.4%).

Finally, surface complexity had a consistent and positive effect on the settlement choice of older plantigrades (38 days post fertilisation) (*F_(2,18)_*  = 54.17, *P*<0.001; Figure 3C). The lowest number of plantigrades settled on smooth polypropylene (0.4±0.2%) and settlement increased with increasing complexity to 46.1±4.3% on the rope. Overall, fewer mussels were suspended in the water column (13.6±2.4%) in comparison to the number of mussels settled on the glass beaker (38.7±3.5%).

### Micro spatial scale settlement onto ropes under hatchery conditions

#### Rope samples in small-scale nursery tanks

**Figure 4:**
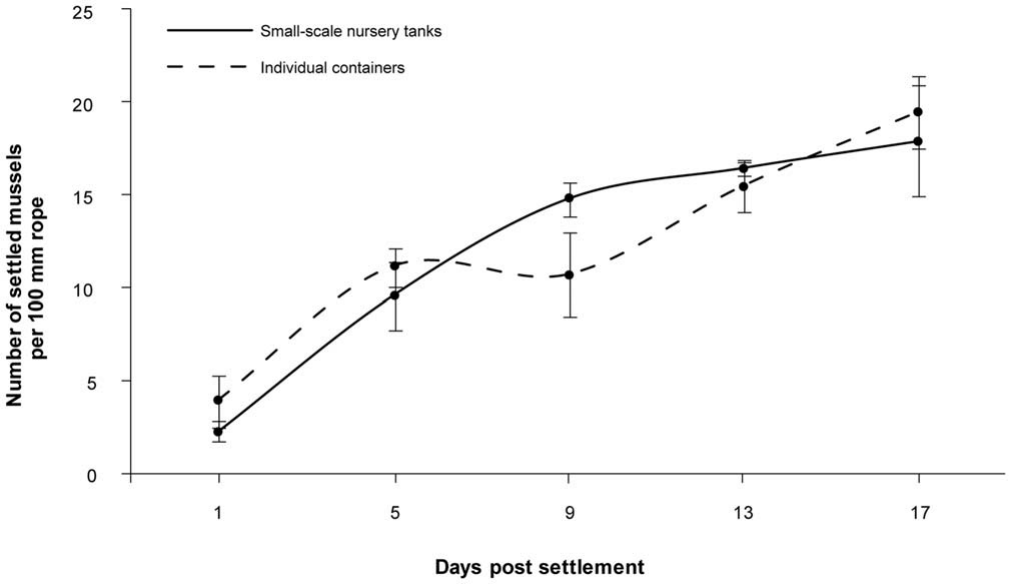
Settlement on the outside of ropes. Mean number of settled mussels on 100 mm long rope samples maintained in small-scale nursery tanks (solid line) and individual containers (dashed line) over time (1 to 17 days post settlement, which correspond to 23 to 39 days post fertilisation). Means ± SE are shown (*n* = 3).

The number of mussels appearing on the outside of ropes that were maintained in continuous culture in small-scale outdoor nursery tanks increased significantly with time (two-factor PERMANOVA: *F_(4,30)_*  = 17.45, *P*<0.001; Figure 4, solid line). The increase in numbers was approximately eight fold from one day post settlement (2.2±0.6 individuals) to 17 days post settlement (17.9±3.0 individuals), with the largest increase between days 1 and 5 post settlement (9.6±1.8 individuals). This effect was consistent between ropes excised from culture in outdoor small-scale nursery tanks (this data) and those assayed in individual containers (Figure 4, dashed line; see below).

Size measurements of mussels one day post settlement (23 days post fertilisation) could not be performed as removal off the rope using tweezers was destructive to their soft shells. However, the average length of mussels collected on the settlement day (22 days post fertilisation) was measured as pediveligers suspended in the water column could be pipetted and their mean shell length was 302.8±4.3 µm. After 5 days post settlement, mussels reached a mean length of 374.2±8.1 µm and their length increased with age to 540.9±12.7 µm, 723.4±11.6 µm, and 989.8±18.7 µm for 9, 13, and 17 days post settlement, respectively.

#### Rope samples in individual containers

In general, rope samples maintained individually in containers (Figure 4, dashed line) showed a similar result in the number of mussels on ropes maintained in small-scale nursery tanks (Figure 4, solid line; see above). Assays of ropes in individual containers further ensured no settlement on the ropes, confirming all subsequent mussels are a product of the initial settlement of pediveligers at 23 days post fertilisation. It also ensured no migration between ropes occurred. The number of mussels on the outside of ropes increased with time, and the time period had a significant effect on the number of individuals (two-factor PERMANOVA: *F_(4,30)_*  = 7.03, *P*<0.001). However, the origin (tank) of the rope sub-samples also had a significant effect on the number of *M. galloprovincialis* (*F_(2,30)_*  = 2.47, *P* = 0.039). The lowest number of mussels were present on the outside of ropes one day post settlement (3.9±1.4 individuals) and increased nearly 3 fold within 4 days (5 days post settlement: 11.1±1.1 individuals). After 9 days post settlement, the number of mussels on the outside of rope decreased slightly (10.7±2.3 individuals) and then increased consistently over time, with higher numbers after 13 days (15.4±1.4 individuals) and 17 days post settlement (19.4±2.0 individuals).

The mean length of mussels maintained in individual containers were similar to individuals grown in small-scale nursery tanks, with 531.9±14.9 µm, 714.0±14.2 µm, and 946.0±15.9 µm for 9, 13, and 17 days post settlement, respectively.

#### Imaging micro spatial habitat with X-ray µCT

**Figure 5:**
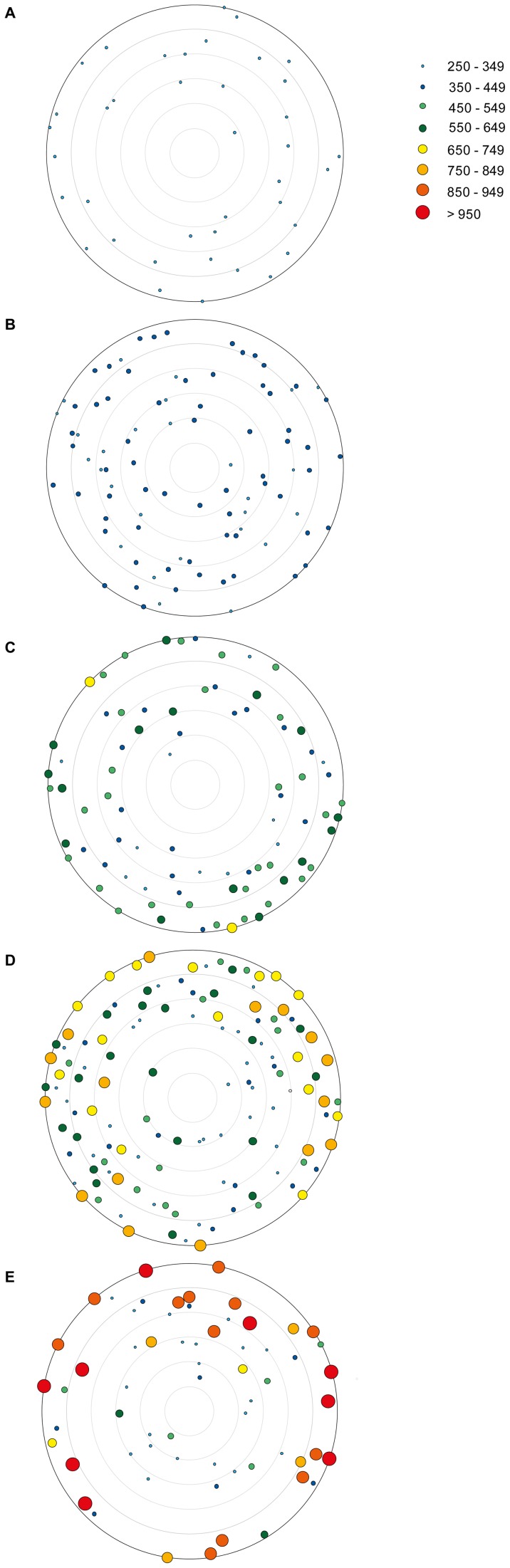
Schematic diagram to illustrate micro spatial scale settlement of individual mussels on and within ropes. (A) 1 day, (B) 5 days, (C) 9 days, (D) 13 days, and (E) 17 days post settlement. The size classes of mussels (250–349, 350–449, 450–549, 550–649, 650–749, 750–849, 850–949, and >950 µm in shell length) are indicated by different colours. The depth of penetration was identified using X-ray µCT and each schematic summarises the overall settlement on all three analysed ropes, therefore representing an overall rope length of approximately 105 mm.

Pediveligers of *M. galloprovincialis* initially settled inside ropes and smaller size classes were deeper inside the ropes over time (Table 1). Notably, there is a clear change in location of mussels from the interior to the exterior of the rope as they increase in size over time (Figure 5A–E). Furthermore, not all mussels grew rapidly and more than 30% of mussels in samples 17 days post settlement were ≤349 µm in shell length.

**Table 1:** Mean distance (µm) of individual mussels to the exterior of ropes after 1, 5, 9, 13, and 17 days post settlement.

	1 day post settlement		5 days post settlement		9 days post settlement		13 days post settlement		17 days post settlement	
Mussel size class (µm)	Distance (µm) ± SE	No of individuals	Distance (µm)± SE	No of individuals	Distance (µm) ± SE	No of individuals	Distance (µm) ± SE	No of individuals	Distance (µm) ± SE	No of individuals
**250–349**	1308.5±165.1	(39)	1949.3±158.4	(27)	1588.0±226.1	(13)	2072.7±245.7	(35)	2770.8±108.5	(22)
**350–449**			1715.2±194.3	(63)	1797.6±59.1	(22)	1442.3±90.9	(18)	1983.0±874.6	(8)
**450–549**					710.3±250.2	(30)	1489.0±149.3	(17)	1668.8±821.6	(5)
**550–649**					847.0±82.2	(16)	1648.9±438.8	(22)	1649.4 ±1508.0	(2)
**650–749**					0.0±0.0	(2)	666.5±347.2	(17)	1747.7 ±1467.3	(2)
**750–849**							469.1±261.2	(16)	1094.5±579.2	(4)
**850–949**									754.6±201.1	(12)
**>950**									463.5±146.9	(9)
Total No of individuals		(39)		(90)		(83)		(125)		(64)

Mussels are grouped in eight size classes (250–349, 350–449, 450–549, 550–649, 650–749, 750–849, 850–949, and >950 µm in shell length) and the number of individual mussels in each size class is shown. Measurements are based on tomograms obtained using X-ray µCT of three individual rope pieces collected at each time point.

At one day post settlement, all mussels ranged in size between 250–349 µm and were located inside ropes with a mean distance to the exterior of 1308.5±165.1 µm (Figure 5A, Table 1). Within the following 4 days, mussels were located deeper within the ropes. Smaller mussels (250–349 µm) were located deeper within ropes (1949.3±158.4 µm) than larger mussels with shell lengths ≥350 µm (1715.2±194.3 µm), although the difference was not significant (two-factor PERMANOVA: *F_(1,84)_*  = 1.66, *P* = 0.288). The majority of individuals (70%) had shell lengths ≥350 µm (Figure 5B, Table 1).

As the size of mussels increased after 9 days post settlement (Figure 5C, Table 1), smaller mussels (≤449 µm) were twice the mean distance from the exterior (deeper) compared to larger mussels (≥450 µm). Individuals ≤449 µm had a mean distance of 1588.0±226.1 µm from the exterior, while individuals in the size classes of 450–549 µm and 550–649 µm had a mean distance from the exterior of 710.3±250.2 µm and 847.0±82.2 µm, respectively. The largest mussels after 9 days post settlement had shell lengths ≥650 µm and were on the outside of the rope samples (0 µm distance). There was no statistically significant effect of the size of mussels on their location at 9 days post settlement (*F_(4,70)_*  = 3.41, *P* = 0.059).

In contrast, the size of mussels significantly affected the depth of location within the rope at 13 days post settlement (*F_(5,107)_*  = 4.31, *P* = 0.015), as well as the tank from which the rope samples were collected (*F_(2,107)_*  = 3.31, *P* = 0.005). Mussels ≤349 µm had the deepest location within ropes with mean distance of 2072.7±245.7 µm from the exterior (Figure 5D, Table 1). Mussels in the size classes of 350–449 µm (*t* = 2.64, *P* = 0.008) and 450–549 µm (*t* = 2.45, *P* = 0.039) were significantly deeper within ropes compared to larger mussels (750–849 µm) with a mean distance of 1442.3±90.9 µm and 1489.0±149.3 µm from the exterior, respectively. Mussels of the largest size class (750–849 µm) were 469.1±261.2 µm from the exterior.

The largest variation in size classes occurred for rope samples 17 days post settlement (Figure 5E, Table 1). The micro spatial scale settlement within ropes was significantly affected by the size of mussels (*F_(7,47)_*  = 2.34, *P* = 0.041) and generally, the distance from the exterior of ropes decreased with increasing shell length. Small mussels (250–349 µm) settled approximately 6-fold deeper within ropes than mussels ≥950 µm (*t* = 5.67, *P* = 0.016) at a depth of 2770.8±108.5 µm from the exterior compared to 463.5±146.9 µm. Overall, more than 30% of all mussels had shell lengths ≤349 µm at 17 days post settlement and were twice as deep within the rope compared to one day post settlement.

## Discussion

*M. galloprovincialis* has clear settlement preferences for more complex materials and high selectivity of settlement sites relative to the size of the individual, from the pediveliger stage through to the plantigrade stage (post metamorphosis). This is the first study to map the three dimensional settlement preferences of mussels on culture rope and subsequently map their locations as individuals grow larger within complex materials, under commercial hatchery operating conditions. Mussels are initially located within ropes and location is significantly affected by the size of the mussel. In general, the distance from the exterior of ropes decreased with increasing shell length. In contrast, distance from the exterior of the rope increased for the smallest size classes with small mussels being deeper inside the ropes with time.

In general, textured and complex surfaces provide an attractive substrate for settlement by offering an increased surface area and protection [8], [31], [32], which in turn reduces post-settlement mortality [8], [21]. This is especially important for pediveligers undergoing metamorphosis, as they do not feed and rely upon stored nutrients and energy while the adult gill/palp feeding mechanism is developed [15]. Imaging of settlement locations at a micro spatial scale using X-ray µCT showed pediveligers initially settle within ropes, which offers protection during this vulnerable stage. These results represent a logical explanation for the high selectivity of pediveligers in their preference of settlement surfaces [6], [8], [9], [14]. While approximately 50% of pediveligers settled on braided polypropylene rope within 48 h, less than 8% settled on textured polypropylene, and less than 1% on smooth polypropylene with no choice given between the test surfaces. Pediveligers of *Mytilus* have the ability to delay metamorphosis for several weeks [16], which facilitates high selectivity of settlement substratum and highlights the importance of complex materials as a secure place for metamorphosis. The absence of favourable substrata, such as fine-branching and filamentous materials [23], [33], prolongs the larval stage of mussels and delays metamorphosis [24]. This is reflected in the results of the assay with no choice between the test surfaces, where the number of unattached planktonic larvae was high for textured and smooth polypropylene, with very low settlement on the latter. This implies that these two surfaces are unfavourable for settlement of pediveligers, although no alternative choice is given and topographic features were present (textured polypropylene). Topographic features are an important settlement cue for pediveligers [19], [20]. However, topography itself is not an exclusive key driver for larval settlement and the scale and level of complexity of the surface plays a key role for settlement as demonstrated in this study.

In contrast to pediveligers, the settlement preference was less pronounced for plantigrades. The direct effect of topography and surface complexity becomes less important as a settlement cue post metamorphosis and metamorphosed individuals will eventually settle on unfavourable substrata when not offered a choice [20], [34], [35].

The settlement of *M. galloprovincialis* was always significantly higher on rope samples than on smooth and textured polypropylene when given a choice between the test surfaces, regardless of age. Pediveligers (22 days post fertilisation) showed the same preference for the most complex surface tested (rope) as plantigrades (30 and 38 days post fertilisation), which underlines the ability to differentiate between favourable and unfavourable surfaces [15] and to make an active settlement choice [33]. There was no change in surface preference with increasing size and ontogenetic stage. This may reflect the range of complexity and size within the rope structure, as opposed to a single texture feature. Overall, ropes are highly effective settlement surfaces for *M. galloprovincialis* providing protected settlement sites inside of the multifilament structure. The distance of pediveligers from the exterior of ropes increased from 1 to 5 days post settlement and decreased afterwards as mussels grew larger, indicating an initial inwards, and subsequent outwards migration. This is in accordance with the observed time period needed for pediveligers to complete metamorphosis. In general, pediveligers of *Mytilus* metamorphose within 1 to 3 days after the first secretion of byssal threads depending on temperature [15]. A sharp increase in food consumption was noticeable 4 days post settlement in this study, demonstrating that the majority of mussels completed metamorphosis within this time period and had begun active feeding. While the inside of ropes offers protection for pediveligers, food availability and space may become limiting factors post-metamorphosis as the size of individuals increase. Consequently, mussels can move outwards as their shell lengths increases as quantified using optical microscopy and X-ray µCT imaging providing improved access to food.

While optical microscopy can determine the number of settled mussels on the outside of ropes, X-ray µCT completes the picture and was used for the first time as a tool to locate individual mussels on and within ropes. As a result, X-ray µCT showed a high proportion of small mussels within the ropes over time, even at 17 days post settlement, which could not be determined using microscopy. In contrast to mussels with increasing shell length, mussels not growing and remaining within their size class are deeper inside the rope over time. Mussels ≤349 µm doubled their penetration depth within 16 days, whereas growing mussels were increasingly located at the exterior of ropes. While X-ray µCT cannot determine whether visualised individuals are alive, the change of the mean distance from the exterior towards the interior strongly supports that pediveligers that have not metamorphosed remained alive and are mobile up to the completion of the study at 17 days post settlement. Overall, X-ray µCT proved to be an excellent non-destructive technique for mapping attachment sites of individuals as early as one day post settlement and represents a powerful tool to understand the settlement ecology and preferences of mussels and other bivalves.

## Conclusions

In conclusion, more complex surfaces are preferred settlement sites with complexity being a key factor in the active selection of sites for settlement, metamorphosis and subsequent location at a micro spatial scale. Pediveligers and metamorphosed plantigrades preferentially settled onto ropes, which represent the most complex tested surface. Small mussels initially settle within ropes (>1000 µm deep) and their depth of penetration in ropes increased with time for the smallest size classes. In contrast, larger mussels were located closer to the exterior of ropes with time. Overall, this study identifies a key driver for settlement selection to support the development of new technologies to manage the settlement and retention of mussels for the aquaculture industry.
